# The cell envelope of *Mycobacterium abscessus* and its role in pathogenesis

**DOI:** 10.1371/journal.ppat.1011318

**Published:** 2023-05-18

**Authors:** Shweta Parmar, Elitza I. Tocheva

**Affiliations:** Department of Microbiology and Immunology, University of British Columbia, Vancouver, Canada; University of Basel, SWITZERLAND

## Abstract

*Mycobacterium abscessus* is a nontuberculosis mycobacterium (NTM) that has shown an exponential rise in its ability to cause disease. Due to its ubiquitous presence in the environment, *M*. *abscessus* is widely implicated in secondary exacerbations of many nosocomial infections and genetic respiratory disorders, such as cystic fibrosis (CF). Contrary to other rapidly growing NTMs, the cell envelope of *M*. *abscessus* harbors several prominent features and undergoes modifications that are responsible for its pathogenesis. Compositional changes of the mycobacterial outer membrane (MOM) significantly decrease the presence of glycopeptidolipids (GPLs) and enable the transition from a colonizing, smooth morphotype into a virulent, rough morphotype. The GPLs are transported to the MOM by the Mycobacterial membrane proteins Large (MmpL), which further act as drug efflux pumps and confer antibiotic resistance. Lastly, *M*. *abscessus* possesses 2 type VII secretion systems (T7SS): ESX-3 and ESX-4, both of which have recently been implicated in host–pathogen interactions and virulence. This review summarizes the current knowledge of *M*. *abscessus* pathogenesis and highlights the clinically relevant association between the structure and functions of its cell envelope.

## Introduction

*Mycobacterium abscessus* is an emerging pathogen that has changed the narrative for rapidly growing nontuberculosis mycobacteria (NTMs) [1–3]. Rapidly growing NTMs do not typically have pathogenic features (i.e., the ability to cause disease); however, *M*. *abscessus* can cause diseased states that, in many ways, resemble those of *M*. *tuberculosis* [4–6]. Classified as a Biosafety Level 2 pathogen, it is the causative agent of various nosocomial and mucocutaneous infections. In humans, *M*. *abscessus* mainly colonizes epithelial cells but can also infect macrophages and neutrophils. Though no specific environmental reservoir has been identified, *M*. *abscessus* can survive and proliferate successfully in amoeba [5,7]. Its exceptional multidrug resistance and ubiquitous environmental presence make it a persistent pathogen that is difficult to treat with standard antibiotics, especially in patients with respiratory disorders and compromised immune systems [8]. Due to increased infectivity of people with underlying pulmonary disorders, *M*. *abscessus* plays a significant role in exacerbating life-threatening genetic disorders including bronchiectasis, chronic obstructive pulmonary disease (COPD), and cystic fibrosis (CF) [8]. Over the years, secondary infections caused by *M*. *abscessus* have surpassed those of common CF-associated pathogens such as *Pseudomonas aeruginosa* and *Burkholderia cepacia* [2,9], making it an important pathogen to consider during disease progression and treatment. In addition to the lungs, *M*. *abscessus* can infect other major organs, including eyes, brain, and skin [1]. Its pervasive nature makes it a fairly common NTM among various skin and soft tissue infections. These infections can spread directly through contaminated water, surgical tools, and other shared materials. Surgical wounds, communal spas and hot tubs, and cosmetic procedures are also common routes for the spread of *M*. *abscessus* skin infections, which include various skin lesions, erythematous nodules, abscesses and sinuses, erythematous papules, and others [1,10]. Ocular *M*. *abscessus* infections mainly cause scleritis, keratitis, and endophthalmitis [11]. A relatively uncommon occurrence of *M*. *abscessus* infection is in the central nervous system (CNS) wherein studies in zebrafish embryo have shown specific neurotropism of *M*. *abscessus* [12]. In humans, once acquired, it can lead to cerebral abscesses and meningitis; these effects are more prevalent in immunocompromised patients like those with HIV or undergoing chemotherapy [13].

The cell envelope of mycobacteria is their main distinguishing feature that plays an essential role during pathogenesis. Surface components in *M*. *abscessus* that have not been collectively observed in any other rapid- or slow-growing mycobacterium include distinct surface lipids, drug efflux pumps, and secretion systems. The interplay among all of these components determines cell envelope integrity and function. This review highlights important surface components of *M*. *abscessus* and summarizes their clinical role in the context of CF.

### I. Cell envelope modifications

**Fig 1:**
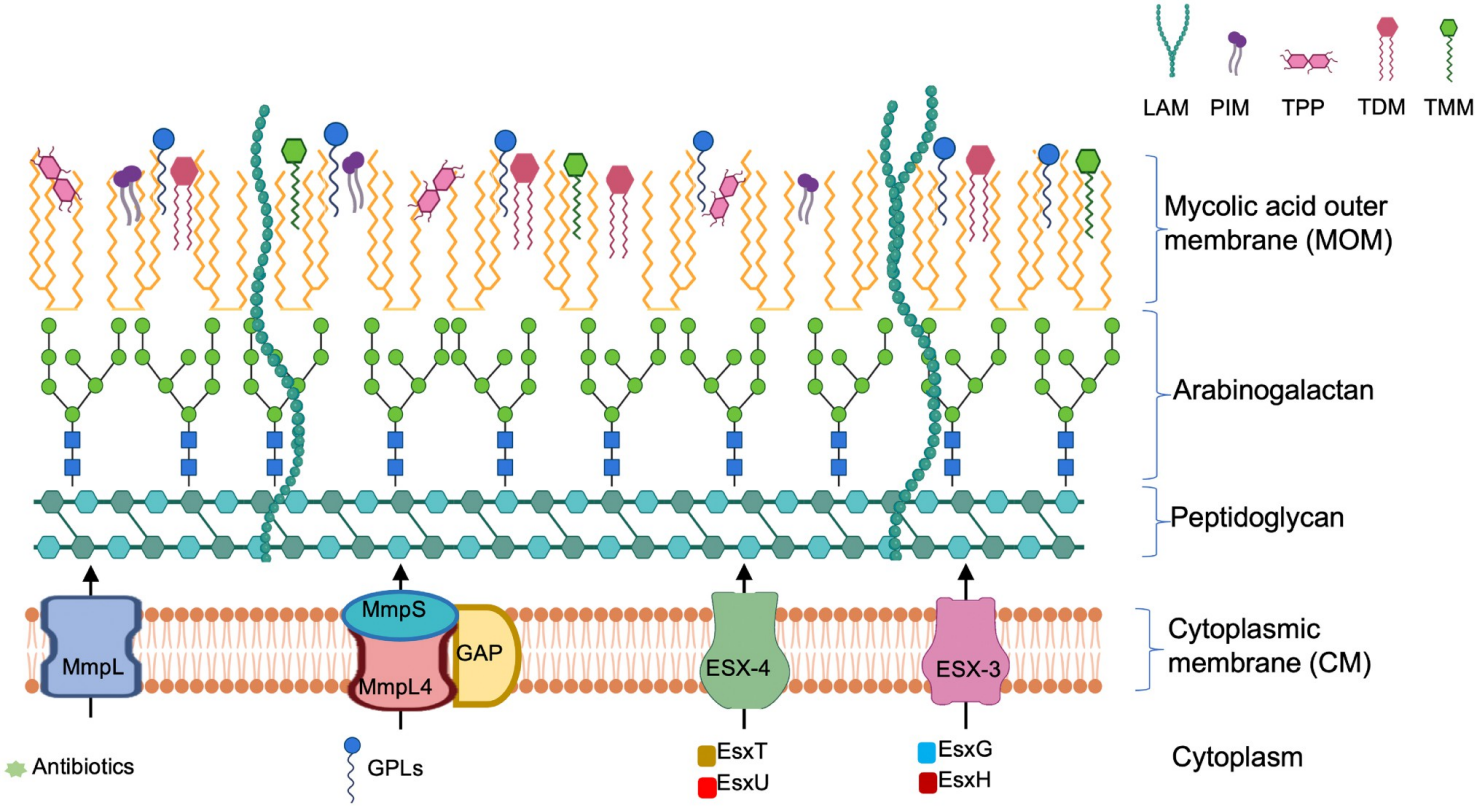
The major components of the *M*. *abscessus* cell envelope. The MOM displays morphologically and immunologically important lipid moieties such as GPLs, TDMs, TMMs, PIMs and TPPs. The CM displays membrane embedded protein assemblies that are essential for the transport of these lipids as well as for the release of virulence factors. These include 3 major complexes: (1) MmpL4-MmpS-GAP, which aid in the transport of GPLs to the outer surface of *M*. *abscessus* and regulate the smooth to rough morphotype transition; (2) MmpLs, implicated in drug efflux mechanisms; and (3) ESX-3 and ESX-4, implicated in infecting host cells through the release of the effector molecules EsxG/H and EsxT/U, respectively. CM, cytoplasmic membrane; GPL, glycopeptidolipid; MmpL, Mycobacterial membrane proteins Large; MOM, mycolic acid outer membrane; PIM, phosphatidyl-myo-inositol dimannoside; TDM, trehalose-6,6-dimycolate; TMM, trehalose monomycolate; TPP, trehalose polyphleate.

Unlike typical cell envelope architectures of gram-positive (monoderm) and gram-negative (diderm) bacteria, the mycobacterial cell envelope has distinct features and composition [14,15]. Mycobacteria belong to the phylum Actinobacteria, typically characterized as monoderm with high guanine and cytosine content in their DNA [16]. Conversely, in addition to their inner/cytoplasmic membrane (CM) and peptidoglycan (PG), Mycobacteria have a unique mycolic acid outer membrane (MOM) and an arabinogalactan layer (Fig 1) [15]. Modifications and up-regulation of clinically important lipid components of the MOM such as glycopeptidolipids (GPLs), trehalose-6,6-dimycolate (TDM), trehalose monomycolate (TMM), trehalose polyphleates (TPPs), and phosphatidyl-myo-inositol dimannoside (PIM) have been documented during pathogenesis [14,17].

**Fig 2:**
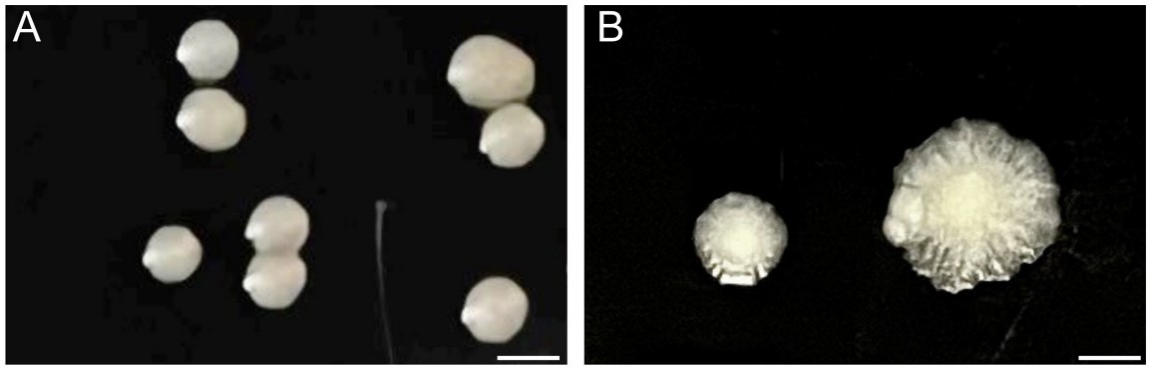
Colony morphotypes in *M*. *abscessus*. (**A**) Smooth colony morphotype, exemplified by the round colony edges, and (**B**) rough colony morphotype, characterized by the irregular edges and flat surface. Freezer stocks of *M*. *abscessus* ATCC 19977 were first used to inoculate liquid Middlebrook 7H9 media supplemented with 0.05% Tween-80, 0.2% glycerol, and OADC and grown at 37°C until mid-log phase (OD_600_ of 0.6–0.7). Cultures were then plated on Middlebrook 7H10 solid media supplemented with 0.2% glycerol and OADC for 5–7 days at 37°C. Scale bar, 3 mm.

Modifications in the cell envelope of *M*. *abscessus* results in the formation of 2 distinct colony morphotypes: rough and smooth (Fig 2) [18–20]. These morphotypes have unique properties affecting bacterial adhesion and host interactions in vivo and in vitro: The smooth morphotype is considered noninvasive, whereas the rough morphotype is virulent and associated with disease progression [19,21]. During infection of host cells, *M*. *abscessus* can transition from smooth into a rough morphotype by modulating GPL levels on its MOM [5,18,19]. The presence of GPLs on the surface of smooth variants makes them less hydrophobic, which is thought to promote sliding motility on agar, as well as induce host colonization upon infection [5,19,22]. The smooth variants also form smaller clumps, which are readily engulfed and lead to faster fusion with lysosomes [5]. The lack of GPLs in rough variants, on the other hand, induces aggregation and cording [12], making it difficult for immune cells to engulf the bacteria and contain the infection [5,6].

Mycobacterial membrane proteins large/small (MmpL/MmpS) and other accessory proteins, such as GPL-addressing proteins (GAPs), form transport assemblies across the CM. Dysfunction in any of these membrane proteins results in defective lipid transport to the cell surface and altered cell morphotype. For example, *M*. *abscessus* strains with irreversible mutations in the genes of mycobacterial nonribosomal peptide synthetases (responsible for GPL synthesis) and deletion of the MmpL4 genes *mmpl4a* or *mmpl4b* (responsible for GPL transport) have led to lack of GPLs and resulted in transition into rough morphotypes [23–25]. This is also accompanied by decreased surface colonization of host epithelial cells, increased proliferation in macrophages, and enhanced innate immune responses, all properties associated with the rough morphotype [23,25,26]. MmpL8 is another important protein in this group that is involved in the synthesis of a previously undefined glycolipid glycosyl diacylated nonadecyl diol (GDND), as well as in the interaction with phagocytic host cells [27]. This glycolipid is unique to *M*. *abscessus* and may have a specific role in infection, especially under the regulation of MmpL8 [27]. Thus, the cell envelope composition drives the initial stages of infection such as surface attachment (by the smooth variant) and intracellular survival and proliferation (by the rough variant).

Imaging studies of bone marrow–derived murine macrophages infected with the smooth variant of *M*. *abscessus* revealed a clear zone between the bacterial surface and the phagosome membrane and prevented phagosome maturation and acidification [5]. The rough variants, on the other hand, formed distinct contacts with the phagosome membrane and, in addition, were able to actively replicate inside the phagosomes, eventually causing rupture and cell-to-cell spread of the bacteria [5,28]. Though it remains unclear when the transition from smooth to rough occurs during infection, the loss of GPLs reveals underlying immune-stimulatory molecules that trigger an immune response by the host. For example, PIM_2_ and lipoproteins exposed on the surface of *M*. *abscessus* were shown to activate the Toll-like receptor-2 (TLR-2), leading to an increase in tumor necrosis factor-alpha (TNF-α)-mediated inflammation [29,30]. Similar to *M*. *tuberculosis*, other virulence-associated glycolipids such as TDMs are also expressed on the surface of *M*. *abscessus* and, together with TPPs, are responsible for cording of the rough variant during granuloma formation [31–33]. Recently, in a zebrafish infection model, the rough morphotype caused inflammation by modulating host TNF-α signaling, leading to necrotic granuloma formation that was comparatively less accelerated in smooth morphotypes [34]. Knock-out mutants of TNF signaling genes (*tnfa*, *tnfr1*, and *tnfr2*) reduced the burden of the rough morphotype but increased the proliferation of the smooth morphotype during infection [34]. Overall, the smooth and rough variants show different intra- and extracellular lifestyles, both of which are needed for bacterial survival and invasion of host cells.

### II. Cell envelope–mediated antibiotic resistance

Several features of the mycobacterial cell envelope confer intrinsic resistance to most antibiotics: GPLs in the MOM, biofilm formation, and drug efflux pumps. As discussed above, the lack of GPLs results in enhanced hydrophobicity of the MOM in rough variants. Since β-lactams, as well as the anti-TB drugs INH, EMB, and streptomycin, are hydrophilic, the susceptibility to these therapeutic agents is extremely low. Furthermore, GPLs are associated with inducing biofilm formation, where the extracellular matrix acts as a physical barrier and protects bacteria from antibiotics such as clarithromycin and amikacin [35,36]. A study of patients with COPD identified the presence of smooth and rough variants in biofilms [37,38]. Since the smooth morphotype is rich in GPLs, the presence of the rough morphotype in the biofilm could act as a virulence factor that promotes invasive growth.

Along with cell wall impermeability, active efflux pumps provide resistance by expelling drug molecules that enter the cell [39,40]. Several mycobacterial drug efflux pumps have been identified, including the MmpLs [41]. As such, these proteins belong to a subclass of the Resistance-Nodulation-Cell Division (RND) permeases, and *mmpL* gene orthologs have been characterized for their role in drug efflux and antibiotic resistance in *M*. *tuberculosis* [42,43]. Moreover, *M*. *abscessus* expresses a greater abundance of MmpL proteins compared to most known rapidly growing NTMs, which may explain their higher antibiotic resistance [44]. For example, the TetR transcriptional regulator MAB_2299c controls genes encoding for the MmpS-MmpL efflux pump and mutations in these genes decrease the resistance of *M*. *abscessus* to clofazimine and bedaquiline [40,45,46]. Lastly, a recent study showed that the GPL-defective mutants of rough morphotype had similar antibiotic susceptibility as the smooth wild-type *M*. *abscessus*, suggesting the involvement of additional factors contributing to resistance during chronic infections [47].

### III. Secretion systems in *M*. *abscessus*

Specialized bacterial secretion systems (type I to type IX) have been recognized as membrane-associated nanomachines that aid in transporting molecules in and out of cells. They promote pathogenesis by secreting virulence factors (substrates) that are required for intracellular survival and for evading the bactericidal mechanisms of the host cell [48]. The type VII secretion system (T7SS) was discovered in *M*. *tuberculosis* and is the major secretion system in mycobacteria [49–51]. There are 5 subtypes of the T7SS known as ESX-1 to ESX-5 [50,51]. Among these, ESX-1, 3, and 5 have been widely studied for their role in mycobacterial survival and pathogenesis [50,52], while limited data are available on the structure and function of ESX-2 and ESX-4. More specifically, ESX-1, with the largest gene locus, supports bacterial survival inside the host, promotes phagosome rupture, and triggers dissemination of mycobacteria through host cell lysis [53,54]. ESX-3 is mainly involved in iron and zinc acquisition and modulation of host cell immunity [55,56]. The ESX-5 secretion system is specific to slow-growing mycobacteria, and, similar to ESX-3, it plays a part in nutrient uptake and immunomodulation [57,58].

**Fig 3:**
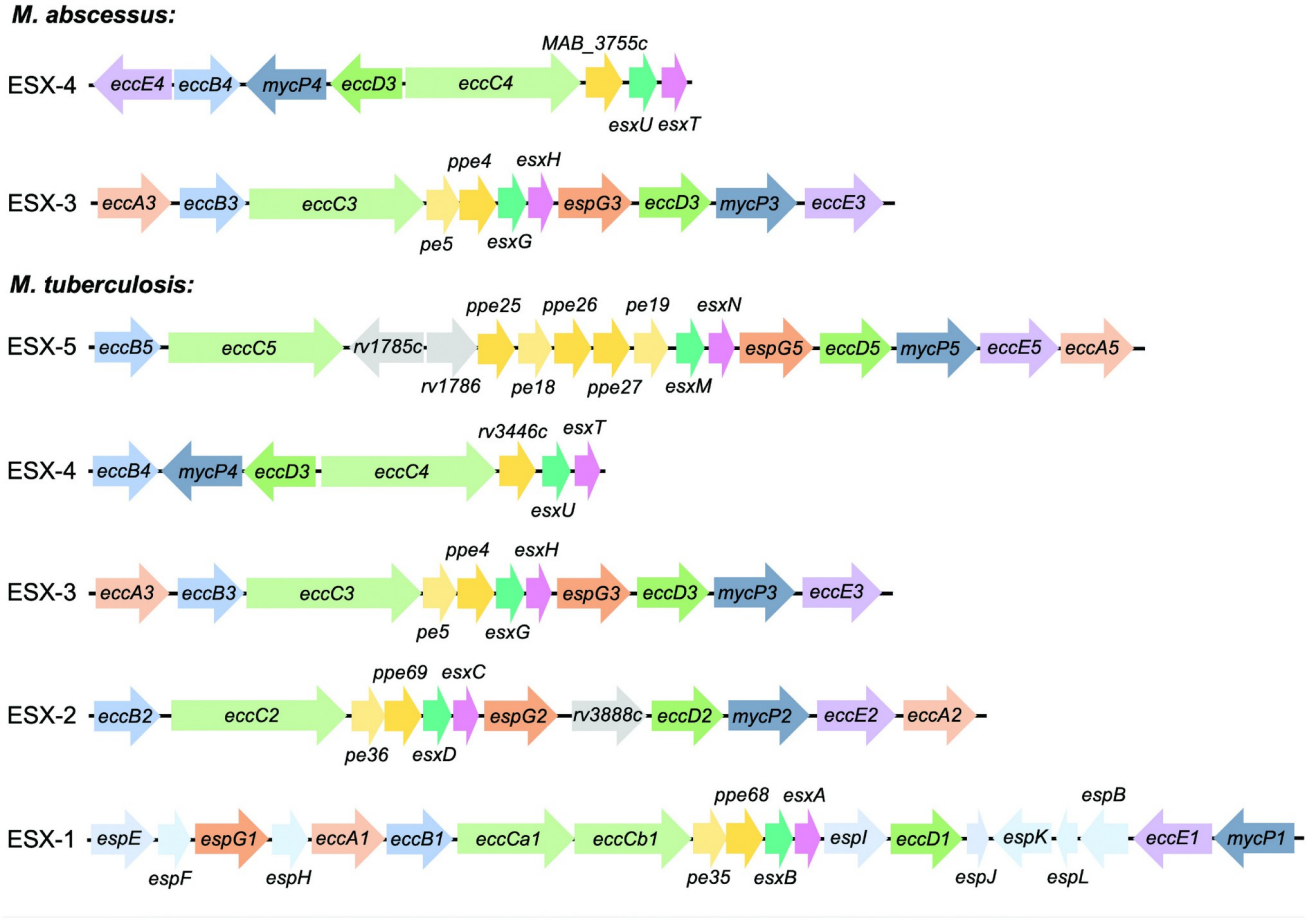
Gene clusters for ESX-3 and ESX-4 in *M*. *abscessus* compared to *M*. *tuberculosis*. Both ESX-3 and ESX-4 encode for major structural components as well as secreted substrates. The genes *eccB*, *eccC*, *eccD*, *eccE*, and *mycP* encode for proteins that make up the intact machinery of the ESX systems. ESX substrate molecules include PE5-PPE4, EsxG, and EsxH for ESX-3, and EsxT and EsxU for ESX-4.

**Fig 4:**
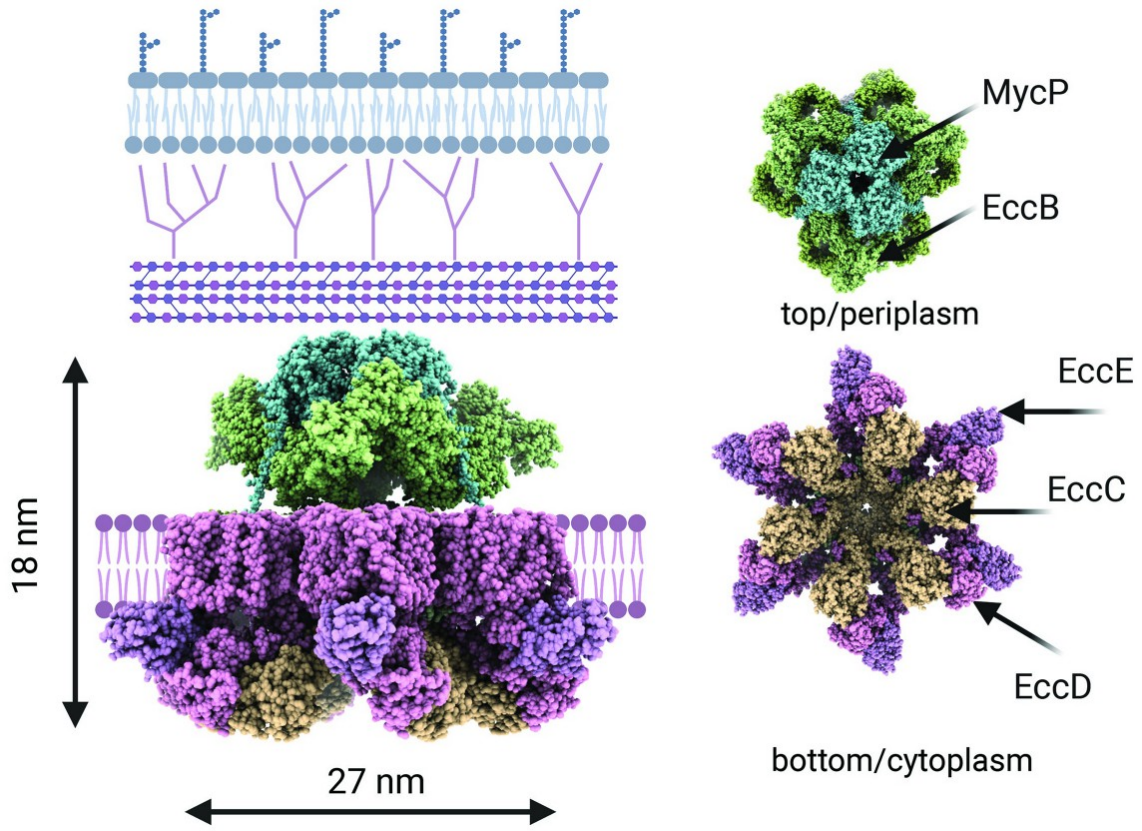
Single-particle cryo-EM structure of the core complex of ESX-5 from *M*. *tuberculosis*. The core complex is composed of EccB, EccC, EccD, EccE, and MycP and spans the CM. Top and bottom views of the complex reveal the multimeric nature of the complex with its dimensions.

Some pathogenic strains of mycobacteria such as *M*. *tuberculosis* have all 5 ESX secretion systems, whereas *M*. *abscessus* possesses only ESX-3 and ESX-4 (Fig 3) [59]. The core complex of all subtypes is thought to be conserved and structural studies of ESX-5 from *M*. *tuberculosis* revealed that it spans the CM and is formed by EccB, EccC, EccD, EccE, and MycP (Fig 4) [60,61]. Briefly, EccB anchors the system within the periplasmic space, EccC encodes for membrane-anchored FtsK–SpoIIIE family ATPase, EccD likely forms a membrane channel, EccE is a membrane and cell wall associated protein, and MycP is a membrane-anchored serine protease. In addition, a cytosolic ATPase, EccA, is required for specific substrate secretion and is recruited to the ESX secretion machinery upon substrate binding [50,62,63]. The known substrates of these ESX systems are broadly divided into 3 types: Esx group that belong to the WXG-100 protein family (e.g., EsxA, EsxB, EsxT, and EsxU); Esp or the ESX-1 secretion-associated proteins (e.g., EspA, B, and C); and the PE-PPE group, which are proteins that have characteristic N-terminal motifs of Pro-Glu and Pro-Pro-Glu (e.g., PE4–PPE5) [50,51]. Upon secretion, these substrates carry out specific functions that aid in bacterial survival and mediate virulence in host cells.

#### ESX-4 and its substrates in *M*. *abscessus*

In most mycobacteria, the *esx-4* cluster lacks the gene for EccE resulting in architectural instability and functionally incapacitated ESX-4 [64]. However, the *esx-4* gene cluster in *M*. *abscessus* encodes for the missing EccE_4_ core complex protein and is essential for survival inside macrophages and amoebae (Fig 3) [4]. Another crucial core component gene is the *eccB4* that affects intracellular survival and modifies the phagosome environment, causing phagosome rupture inside host cells [4]. The effects of ESX-4 are known to be substrate dependent since deletion of ESX-4 core complex genes results in lower expression of EsxU and EsxT (ESX-4 substrates) as well as PE-PPE proteins (ESX-3 substrates) [4]. In line with these findings, a recent study shows that EsxU and EsxT from *M*. *abscessus* form a heterodimer involved in phagosome membrane damage of macrophages [65]. Therefore, the EsxU/EsxT pair is crucial for inducing membrane permeability, which is advantageous during early stages of bacterial infection. However, when tested in vivo in zebrafish and mice, knockout mutants of EsxU/EsxT induced hypervirulence via increased bacterial growth and granuloma/abscess formation [65]. These findings from 2 different experimental settings imply that the ESX-4 substrates may be essential during the initial stages of infection and down-regulated subsequently to induce hypervirulence and spread of the infection. Since ESX-4 induces pathogenicity in *M*. *abscessus* in a manner similar to ESX-1 in *M*. *tuberculosis*, it may act analogously to the ESX-1.

#### ESX-3 and its substrates in *M*. *abscessus*

The ESX-3 secretion system is the most conserved among all T7SSs. The functional and structural characteristics of ESX-3 have been widely studied in *M*. *tuberculosis*, revealing a role in maintaining intracellular survival in macrophages, delaying phagolysosome fusion, and acquiring bacterial nutrients during macrophage infection [50]. The genes for ESX-3 are expressed in response to iron limitation and maintain metal homeostasis in mycobacteria [66–68]. Along with the main core components of ESX-3, the operon encodes for various ESX-3 substrates including the EsxG, EsxH along with PE5-PPE4 proteins (Fig 3). EsxH, in particular, plays a key role in *M*. *tuberculosis* pathogenicity by impeding the hepatocyte growth factor receptor substrate (HRS) component of the human endosomal sorting complex required for transport (ESCRT) pathway, which is essential for endomembrane repair [55,56,69]. In *M*. *tuberculosis*, the presence of both iron and zinc strongly represses *esx-3* expression, leading to lower secretion of EsxG and EsxH, and ultimately diminishing intracellular survival in macrophages [68]. EsxG and EsxH also interfere with lysosomal trafficking and phagosome maturation [55,56,69]. The secretion of these 2 proteins directly correlates with intracellular growth and bacterial virulence, and mutants lacking these substrates are highly attenuated for growth in human macrophages [70]. Furthermore, expression of recombinant ESX-3 substrates PE5 and PE15 in *M*. *tuberculosis* and *M*. *smegmatis* has been shown to improved intracellular survival and alter the innate immune responses by reducing transcription levels of pro-inflammatory cytokine IL-12 and up-regulating the levels of the anti-inflammatory cytokines IL-10, IL-4, IL-5, and TGF-β [70].

Similar to *M*. *tuberculosis*, the ESX-3 of *M*. *abscessus* has been shown experimentally to play a role in pathogenesis of mice [71]. Mutations in the genes for EsxG and EsxH induce less systemic and local inflammatory responses compared to wild-type bacteria during infection [71]. In *M*. *abscessus*, ESX-3 induces pro-inflammatory cytokines like TNF-α, IL-6, IL-1β, and IL-12p40 in murine and human macrophages and is associated with activation of mitogen-activated protein kinase (MAPK) as well as NF-κB signaling after infection [71]. Deletion mutants of the ESX-3 result in decreased production of inflammatory cytokines, lowered levels of cyclooxygenase 2 (COX-2) and nitric oxide synthase (iNOS), and reduced neutrophil recruitment to lung tissues [71]. Although the prospects of ESX-3–mediated pathogenic functions in *M*. *abscessus* are intriguing, more studies are required.

### IV. Interplay between major cell envelope components in *M*. *abscessus*

Cell envelope dynamics affect membrane morphology and have functional implications for *M*. *abscessus* pathophysiology. It is therefore important to consider interactions between various cell envelope components. For example, the transition from smooth to rough morphotype allows *M*. *abscessus* to evade the host’s immune system. On the other hand, the ESX systems induce pathogenicity by secreting effector molecules. In order to cross the thick cell envelope, changes in the lipid and protein composition of the CM and MOM are required. This raises the question of whether the change from the smooth to rough morphotype affects the expression and function of the ESX secretion systems. Since the transport of membrane lipids is dependent on CM proteins such as MmpLs, their expression may also affect the synthesis of ESX-3 and ESX-4. Since the function of ESX-3 and ESX-4 is not well characterized, it is also possible that effector substrates alter the expression and/or transport of MOM lipids through the MmpL protein complexes in order to promote their own secretion. Association between different cell envelope components was presented in *M*. *marinum* and *M*. *tuberculosis* wherein ESX-1 acts in concert with the membrane lipid PDIM during phagosomal damage [54,72]. In context to the similarity between ESX-1 and ESX-4, it was also suggested that ESX-4 may have a similar combined effect with other membrane assemblies like the MmpL8 that transports GDND in order to mediate its effects inside the host cell [51].

In addition to their role during pathogenesis, secretion systems have been shown to affect membrane properties such as capsule integrity, hydrophobicity, and biofilm formation. In slow-growing mycobacteria, the ESX-5 system is involved in maintaining capsule integrity and surface hydrophobicity through the secretion of PPE-10 substrate [73]. Studies on the ESX-5 system in *M*. *marinum* lacking the MOM porin MspA have demonstrated impaired bacterial growth, suggesting a link between ESX-5 and membrane permeability [57]. Deletion of the ESX-1 substrates in *M*. *marinum* causes attenuation of biofilm formation and sliding motility [74], and impaired ESX-3 in *M*. *marinum* results in decreased permeability, abnormal colony morphology, and reduced biofilm formation [75]. Since *M*. *abscessus* encodes for ESX-3 and ESX-4 only, understanding how these 2 secretion systems coordinate their activity to compensate for the lack of ESX-1 and ESX-5 is of major significance.

### V. Clinical implications of *M*. *abscessus* in CF

The distinct cell envelope profile of *M*. *abscessus* makes it a highly potent infectious agent in many mucocutaneous disorders. Along with a high level of resistance to multiple antibiotics, *M*. *abscessus* is also resistant to most disinfectants and, hence, responsible for various postsurgical infections [1,76]. The nature of infection is generally progressive, but a fulminant course has also been observed, especially in acute respiratory disorders [76,77]. Overall, *M*. *abscessus* is proving to be one of the most worrisome NTMs prevalent in present times [2,78,79].

CF is an autosomal recessive disorder with a dysfunctional CF transmembrane conductance regulator (CFTR) protein, mainly affecting the respiratory tract. This life-threatening disorder is globally prevalent with a high incidence rate among white populations, especially in children [80]. *M*. *abscessus* has emerged as a major pathogen in CF patients, leading to further deterioration of lung function [2]. A defective CFTR protein affects chloride and sodium ion levels inside airway epithelial cells, lowering the water content in the mucus and impairing proper airway secretion. The thicker mucus leads to inefficient mucociliary clearance and renders the airway epithelium a favorable environment for bacterial colonization [79].

Membrane dynamics between smooth and rough morphotypes has been proposed as the link between CFTR dysfunction and increased susceptibility to *M*. *abscessus* [81,82]. The smooth morphotype colonizes the airway epithelium and the transition into the rough morphotype induces virulence in CF patients [83,84]. The smooth variant is predominant during the initial phases of infection when its GPL-abundant MOM permits evasion of the host’s immune system by masking the bacterial cell surface from immune instigative molecules such as TLR-2 [85]. This masking prevents downstream signaling via IL-8 and the recruitment of neutrophils [85]. Once inside the host, the invasive role of the rough variants is dominated by their cord-forming ability as shown in the CF model organism zebrafish [86]. In support of this model, rough variants are routinely isolated from samples of chronically ill patients [19,87,88]. Even though CF is a genetic disorder, it does provide a conducive environment for pathogens, which need to be treated with antibiotics. In addition to the hydrophobic MOM and MmpLs efflux pumps discussed here, *M*. *abscessus* has an unusually high resistance to most antibiotics due to various antibiotic-modifying enzymes [89]. As a result, the inability to treat this infection promotes chronic disease [40,90]. Furthermore, the properties of *M*. *abscessus* biofilms (such as viscoelasticity and stiffness) are comparatively higher than most other pathogenic bacteria and are suggested to hinder airway clearance [91].

**Fig 5:**
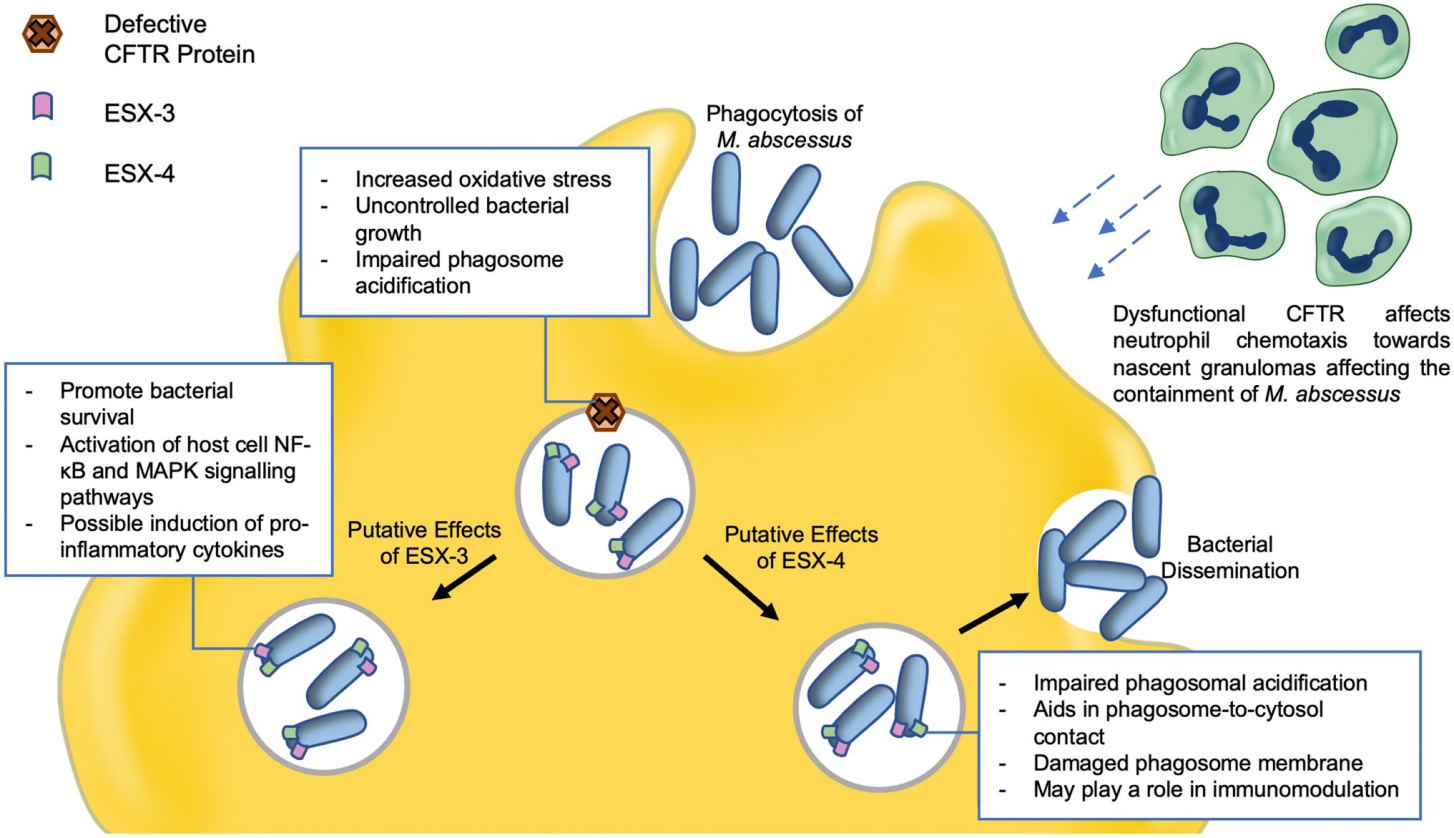
Proposed effects of ESX-3 and ESX-4 in *M*. *abscessus* on CFTR-defective phagocytes. *M*. *abscessus* as an NTM known to exacerbate prerespiratory conditions such as CF through mechanism that are not yet well characterized. The effects of ESX-3 and ESX-4 may overlap with the pathophysiology of a dysfunctional CFTR protein and worsen disease states.

Even though defective CFTR mainly affects alveolar epithelial cells, some ESX-3– and ESX-4–associated cellular changes have also been identified in macrophages [92] (Fig 5). For example, ESX-4 was shown to cause phagosome damage and promote intracellular survival by reducing phagosome acidification [4]. Similarly, one of the effects of a dysfunctional CFTR protein is also the impairment of phagosome acidification [81,82], a process that may be further amplified by the ESX-4. Generally, a defective CFTR leads to high inflammatory responses that progressively deteriorate lung function. This pathophysiology of increased oxidative stress and neutrophil response is also observed in zebrafish with a dysfunctional CFTR protein [93]. When zebrafish with a dysfunctional CFTR were infected with *M*. *abscessus*, lower ROS production was observed, which led to impaired neutrophil chemotaxis, ultimately protecting the bacteria [81,82,94]. The role of ESX-3 during CF is still under investigation.

## Concluding remarks

The wide range of pathogenic factors in *M*. *abscessus* gives this organism an unprecedented capacity to elicit a range of infection patterns. More importantly, most of these pathogenic factors revolve around the cell envelope structure and composition of the bacterium. The flexibility to transform into a more virulent morphotype aids in deceiving the host’s immune system and enables systemic spread. A unique MOM and numerous efflux pumps form a strong barrier against the host’s immune system as well as a wide range of antibiotics. Furthermore, the presence of a distinct ESX-4 provides *M*. *abscessus* with armaments to modulate the host immune system, allowing for a more aggressive progression of the infection. Many of these features remain obscured and require further studies to develop a better understanding of the infection mechanisms employed by this organism.
